# Editorial: Advances in perceptual learning: new directions, techniques, and applications

**DOI:** 10.3389/fnins.2026.1935934

**Published:** 2026-08-11

**Authors:** Qing He, Chang-Bing Huang, Juan Chen, Benjamin Thompson

**Affiliations:** 1 State Key Laboratory of Cognitive Science and Mental Health, Institute of Biophysics, Chinese Academy of Sciences, Beijing, China; 2 State Key Laboratory of Cognitive Science and Mental Health, Institute of Psychology, Chinese Academy of Sciences, Beijing, China; 3 Department of Psychology, University of Chinese Academy of Sciences, Beijing, China; 4 Center for the Study of Applied Psychology, Guangdong Key Laboratory of Mental Health and Cognitive Science, School of Psychology, South China Normal University, Guangzhou, China; 5 School of Optometry and Vision Science, University of Waterloo, Waterloo, ON, Canada; 6 Centre for Eye and Vision Research, Hong Kong, Hong Kong SAR, China

**Keywords:** brain plasticity, brain-computer interface, feedforward and feedback, learning and memory, non-invasive brain stimulation, top-down, visual perceptual learning

For decades, the field of visual perceptual learning (VPL) has been shaped by a foundational puzzle, that is, improvements acquired through practice often fail to transfer even to slightly altered stimulus features or shifted retinal locations (Lu and Dosher, 2022). This specificity has historically been interpreted as strong evidence that VPL occurs in early visual cortices, where neurons possess small receptive fields and sharp selectivity for simple features such as orientation, spatial frequency, and retinal position (Karni and Sagi, 1991; Schoups et al., 1995; Yu et al., 2016; see a review He et al., 2022b). The logic was straightforward: if learning effect is specific, the neural locus of VPL will most likely be early. Notably, concerning the “early stage” hypothesis, beyond the interference from psychophysical evidence, it has also been supported by evidence from both electrophysiological recording in animals and brain imaging in humans. For example, fMRI studies showed that the BOLD signal in V1 changed after VPL (Bao et al., 2010; Ghose et al., 2002; Hua et al., 2010; Maertens and Pollmann, 2005; Sale et al., 2011; Schoups et al., 2001; Yotsumoto et al., 2008). Specifically, the signal in subcortical nuclei of visual information processing, such as the magnocellular layer of the lateral geniculate nucleus (LGN), increased after extensive contrast detection learning (Yu et al., 2016), providing strong evidence that the neural locus of VPL can be located before V1 in humans.

Yet this consensus has been progressively challenged. Paradigms such as double training (Xiao et al., 2008) and reactivation-based learning (Amar-Halpert et al., 2017; Kondat et al., 2025) have demonstrated that learning can indeed transfer across retinal locations and stimulus attributes when training protocols are appropriately designed. These findings forced a reconsideration: specificity may not be an immutable property of VPL, but rather a reflection of the particular mechanisms recruited by a given training regimen. This in turn opened the possibility that VPL can occur at multiple levels of the visual hierarchy also evolved. Similarly, this “late stage” hypothesis is

also supported from both electrophysiological recording in animals and brain imaging in humans. For instance, the lateral intraparietal area (LIP) and anterior cingulate cortex (ACC), the higher-order decision-making regions of the brain, undergo changes associated with VPL, rather than the visual cortex (Kahnt et al., 2011; Law and Gold, 2008).

Building on these findings, different conceptual theories have been proposed, which appear to reconcile the competing early- and late-stage hypotheses. The Reverse Hierarchy Theory (Ahissar and Hochstein, 2004) proposes that VPL is a top-down guided process, beginning at high-level areas and progressing backward to lower levels when finer discrimination is demanded. The Reweighting Theory (Dosher et al., 2013; Dosher and Lu, 1998) posits that learning optimizes the readout of sensory information through reweighting connections between representational and decision layers, with generalization depending on the overlap of reweighted connections. Complementing these, the two-stage model (Shibata et al., 2014) distinguishes between fast, task-related learning and slow, consolidation-dependent learning, each associated with distinct neural substrates. What unites these frameworks is the recognition that higher-order areas, particularly parietal and prefrontal cortices, critically modulate early visual processing through top-down feedback (Jing et al., 2021; Yan et al., 2018). While these feedback signals strongly influence whether and how learning generalizes, accumulating neurophysiological and brain imaging evidence also supports a significant contribution of neural plasticity within or before early visual cortices (e.g., LGN, and V1/V2) to stimulus-specific changes (Bao et al., 2010; Ghose et al., 2002; Yotsumoto et al., 2008; Yu et al., 2016). Therefore, specificity and transfer likely emerge from interactions across the visual hierarchy, rather than being determined solely by higher-order control. It is worth emphasizing that the empirical phenomena reviewed here, including both specificity and transfer, can also be accommodated by computationally explicit, feature-invariant, or hybrid learning frameworks. Notably, this observation is consistent with the whole-brain perspective advanced by Maniglia and Seitz (2018), who argue that learning arises from distributed plasticity across multiple brain systems rather than a single locus, and that the degree of specificity or transfer reflects how training conditions and individual factors modulate this distributed pattern. Accordingly, while top-down modulation offers a parsimonious account of many findings, the current evidence remains compatible with multiple theoretical perspectives.

Historically, however, evidence for these accounts has been largely correlational. Neuroimaging and electrophysiological studies have documented training-induced changes in both early sensory and higher-order areas (Chen et al., 2015; Jing et al., 2021; Mukai et al., 2007; Yan et al., 2014), but correlation does not establish causation. A major advance in the field has come from the application of non-invasive brain stimulation techniques, which allow direct causal interrogation of the brain activity underlying learning and transfer. Transcranial magnetic stimulation (TMS) has been used to map how the neural locus of generalization shifts from parietal to occipital cortex after training (Chang et al., 2013), and to demonstrate the causal involvement of parietal cortex in maintaining post-training working memory representations (Jia et al., 2021). Meanwhile, transcranial electrical stimulation (tES), including transcranial direct current stimulation (tDCS), transcranial alternating current stimulation (tACS), and transcranial random noise stimulation (tRNS), has enabled researchers to not only test causal involvement but also to actively facilitate learning by modulating cortical excitability during both training and offline consolidation (He et al., 2021, 2022a, 2023; Yang et al., 2022; Zhu et al., 2024).

Notably, recent studies have begun to identify the specific neural oscillatory mechanisms recruited by tES. Systematic comparisons across different tES stimulation protocols have revealed that VPL can be reliably enhanced by both high-frequency tRNS and alpha-band (10 Hz) tACS applied over the visual cortex, whereas other frequencies (e.g., 20 Hz or 40 Hz) and 10 Hz applied over other cortical regions (i.e., sensorimotor cortex), produce no such benefit (He et al., 2022a, 2023), which suggests that top-down driven alpha oscillations play a causal role in visual plasticity. These findings extend the causal toolkit beyond simple excitability modulation, pointing to specific neural rhythms as potential targets for optimizing learning interventions. However, it is important to note that human tACS studies, while powerful for establishing causal involvement, cannot readily disentangle specific microcircuit mechanisms, such as thalamocortical loops vs. long-range feedback, from broader network-level effects. Moreover, the actually underlying neural mechanisms of alpha tACS-facilitated VPL should be proved by solid electrophysiological and/or brain imaging evidence.

It is also worth noting that effect sizes and reproducibility in neuromodulation studies can vary substantially across laboratories, owing to differences in sample size, stimulation parameters (e.g., intensity, duration, and electrode montage), and individual physiological baselines or state; mitigating this variability therefore necessitates larger-scale, multi-center investigations and the adoption of precision stimulation protocols tailored to individual brain anatomy and functional states.

Against this background, Consorti and Sale provide a comprehensive synthesis that anchors this Research Topic. Their review argues that VPL reflects a dynamic reweighting between bottom-up sensory signals and top-down feedback across the cortical hierarchy. Drawing on evidence from primates to rodents, including recent work showing that suppressing the lateromedial cortex in mice abolishes both acquisition and retention of learned discrimination (Consorti et al., 2024), they make the case that learning is not a localized event but a circuit-wide reorganization. This top-down perspective, they contend, is probably the key to resolving the specificity-transfer tension: early sensory areas encode feature-specific representations, while higher-order areas provide the contextual and task-relevant feedback that determines whether learning generalizes. The convergence of this framework with the oscillatory findings from tACS studies is particularly compelling: alpha-band feedback from higher-order areas may be the very mechanism through which top-down signals sculpt early sensory representations.

While this top-down perspective provides an elegant framework, it should be considered alongside evidence for substantial plasticity within early visual cortices themselves. Studies in humans and animals have demonstrated stimulus-specific physiological changes, such as changed cortical tuning, that are localized to V1/V2 (Hua et al., 2010; Schoups et al., 2001) and enhanced cortico-cortical communications between visual cortex and high-order cortical regions (Chen et al., 2015; Jia et al., 2020). Therefore, a comprehensive account of VPL likely requires integrating both bottom-up sensory encoding and top-down modulatory control.

This theoretical framework finds direct causal support from Jin et al. in this Research Topic. Combining high-frequency tRNS with contour-detection training, the researchers demonstrated a double dissociation: occipital stimulation enhanced overall learning gains, consistent with refinement of early sensory representations, whereas parietal stimulation uniquely promoted transfer across curvatures, supporting the view that higher-order regions are critical for flexible generalization (Jin et al.). Notably, these effects were stage-dependent: parietal stimulation accelerated early acquisition, while occipital stimulation sustained learning during the later asymptotic phase. This temporal dissociation echoes the two-stage model and provides causal evidence that different hierarchical levels contribute differentially across the time course of learning. Importantly, by applying stimulation online during training rather than offline before or after, this study extends the causal evidence from correlational to interventional. Moreover, the transfer of learning induced by parietal stimulation offers a viable strategy to overcome the long-standing “curse” of poor generalization in real-world VPL translational application.

In interpreting the literature linking perceptual learning, attention, and working memory, it is useful to distinguish mechanistic evidence (e.g., animal neurophysiology, causal interventions), behavioral evidence (e.g., psychophysical performance, transfer patterns), and clinical evidence (e.g., rehabilitation outcomes). While these levels of analysis are mutually informative, caution is warranted when extrapolating mechanistic claims directly from behavioral or clinical findings, or *vice versa*.

The top-down framework also extends beyond sensory processing to interactions with cognitive systems. George and Patel show that learned spatial suppression, acquired through statistical regularities in visual search, carries over to impair visual working memory, but only under high memory load. This load-dependent impairment suggests that attentional suppression operates by down-regulating sensory gain at the suppressed location, and that this spatial bias competes for limited cognitive resources. Critically, this finding broadens the scope of top-down modulation beyond the visual hierarchy, demonstrating that history-driven attentional biases can persist and interfere with internal representations—a consequence that modular accounts of statistical learning cannot readily accommodate.

Perhaps the most compelling validation of this hierarchical, plasticity-based framework comes from its clinical translation. Traditional VPL-based rehabilitation, while efficacious in laboratory settings, has faced substantial barriers to clinical adoption (Huang et al., 2008). Negative clinical outcomes likely reflect a confluence of factors, including disease heterogeneity, insufficient training dosage, diverse training regime, and variable patient adherence due to boredom, rather than an intrinsic inefficacy of VPL *per se*.

In the digital era, implementing VPL within virtual reality (VR) or augmented reality (AR) environments offers a promising strategy to circumvent the limitations of conventional VPL-based interventions. Namgung et al. address these challenges by applying dichoptic visual perceptual learning via VR to children with intermittent exotropia. This condition is particularly suited to this approach: traditional non-surgical therapies, such as patching and overcorrecting lenses, primarily rely on monocular use and do not actively promote binocular fusion. In contrast, the VR-based intervention delivers independent stimuli to each eye through a head-mounted display, enabling precise dichoptic training that is calibrated to each child's deviation angle. Critically, the immersive, game-like environment transforms repetitive training into an engaging, interactive experience, thereby addressing the adherence challenge that has plagued conventional protocols. The results are striking: after 8 weeks of home-based training, the odds of achieving sensory fusion increased five-fold in the full cohort and 20-fold in the subgroup with subnormal baseline stereopsis, demonstrating that when combined with appropriate technology, the plasticity mechanisms of VPL can be effectively mobilized for clinical benefit.

To date, the VR/AR-based VPL has been applied to a range of visual disorders, including strabismus, glaucoma, and amblyopia, demonstrating that these technologies enable diversified training formats and effectively improve visual acuity, contrast sensitivity, and spatial integration (Bao and Engel, 2019; Dong et al., 2023). Notably, Gao et al. (2025) recently developed an AR-based visual training system that selectively enhances the function of the damaged parvocellular pathway in amblyopic patients through real-time visual input processing. After training with lightweight AR glasses, both healthy volunteers and amblyopic patients showed significant visual function improvements. This approach represents a fundamental iteration of the traditional VPL framework, AR technology transforms training from a laboratory task into a naturalistic experience embedded in daily life, and its high adherence and efficacy powerfully validate the logic of technology-empowered VPL. As expected, the application of VR/AR-based VPL will benefit a diverse range of clinical conditions, underscoring the broad translational relevance of VR/AR-based digital interventions. These include developmental disorders such as infantile nystagmus syndrome, acquired deficits such as visual field loss following traumatic brain injury or stroke, degenerative conditions such as macular degeneration and glaucoma, and age-related functional declines in low-vision populations (Bao and Engel, 2019; Dong et al., 2023; Gao et al., 2025).

Looking ahead, the review by Consorti and Sale points to critical unanswered questions. How exactly do top-down signals reshape early visual microcircuits, through parvalbumin-positive interneurons, horizontal connections, or both? How do frequency-specific oscillatory entrainment and stochastic resonance mechanisms jointly contribute to learning enhancement? What boundary conditions determine whether learning transfers broadly or remains stubbornly specific, and can neuromodulation be optimized to reliably promote transfer? How can the insights from basic stimulation studies be translated into effective, personalized clinical protocols, and how durable are the gains observed in patient populations? The studies gathered in this Research Topic do not merely answer these questions; they refine them, transforming broad theoretical postulates into testable hypotheses (Consorti and Sale). By combining causal interventions, cognitive frameworks, and clinical translation, they chart a path toward a comprehensive understanding of VPL—one in which specificity and transfer are not opposing forces but complementary outcomes of a dynamic, hierarchical system. We hope this Research Topic fosters continued dialogue between basic and applied research, driving both mechanistic insight and meaningful clinical innovation.
